# Effectiveness of a Multistrategy Behavioral Intervention to Increase the Nutritional Quality of Primary School Students’ Web-Based Canteen Lunch Orders (Click & Crunch): Cluster Randomized Controlled Trial

**DOI:** 10.2196/26054

**Published:** 2021-09-07

**Authors:** Rebecca Wyse, Tessa Delaney, Fiona Stacey, Rachel Zoetemeyer, Christophe Lecathelinais, Hannah Lamont, Kylie Ball, Karen Campbell, Chris Rissel, John Attia, John Wiggers, Sze Lin Yoong, Christopher Oldmeadow, Rachel Sutherland, Nicole Nathan, Kathryn Reilly, Luke Wolfenden

**Affiliations:** 1 School of Medicine and Public Health University of Newcastle Callaghan Australia; 2 Hunter New England Population Health Wallsend, New South Wales Australia; 3 School of Exercise and Nutrition Sciences Deakin University Burwood, Victoria Australia; 4 School of Public Health University of Sydney Sydney Australia

**Keywords:** nudge, choice architecture, intervention, online canteen, online ordering systems, digital interventions, school children, school food service, canteens, menu labeling

## Abstract

**Background:**

School food outlets represent a key setting for public health nutrition intervention. The recent proliferation of web-based food ordering systems provides a unique opportunity to support healthy purchasing from schools. Embedding evidence-based choice architecture strategies within these routinely used systems provides the opportunity to impact the purchasing decisions of many users simultaneously and warrants investigation.

**Objective:**

This study aims to assess the effectiveness of a multistrategy behavioral intervention implemented via a web-based school canteen lunch ordering system in reducing the energy, saturated fat, sugar, and sodium content of primary students’ web-based lunch orders.

**Methods:**

The study used a parallel-group, cohort, cluster randomized controlled trial design with 2207 students from 17 Australian primary schools. Schools with a web-based canteen lunch ordering system were randomly assigned to receive either a multistrategy behavioral intervention that included choice architecture strategies embedded in the web-based system (n=9 schools) or the standard web-based ordering system only (n=8 control schools). Automatically collected student purchasing data at baseline (term 2, 2018) and 12 months later (term 2, 2019) were used to assess trial outcomes. Primary trial outcomes included the mean energy (kJ), saturated fat (g), sugar (g), and sodium (mg) content of student lunch orders. Secondary outcomes included the proportion of all web-based lunch order items classified as *everyday*, *occasional*, and *caution* (based on the New South Wales Healthy School Canteen Strategy) and canteen revenue.

**Results:**

From baseline to follow-up, the intervention lunch orders had significantly lower energy content (−69.4 kJ, 95% CI −119.6 to −19.1; *P*=.01) and saturated fat content (−0.6 g, 95% CI −0.9 to −0.4; *P*<.001) than the control lunch orders, but they did not have significantly lower sugar or sodium content. There was also a small significant between-group difference in the percentage of energy from saturated fat (−0.9%, 95% CI −1.4% to −0.5%; *P*<.001) but not in the percentage of energy from sugar (+1.1%, 95% CI 0.2% to 1.9%; *P*=.02). Relative to control schools, intervention schools had significantly greater odds of having *everyday* items purchased (odds ratio [OR] 1.7, 95% CI 1.5-2.0; *P*<.001), corresponding to a 9.8% increase in *everyday* items, and lower odds of having *occasional* items purchased (OR 0.7, 95% CI 0.6-0.8; *P*<.001), corresponding to a 7.7% decrease in *occasional* items); however, there was no change in the odds of having *caution* (least healthy) items purchased (OR 0.8, 95% CI 0.7-1.0; *P*=.05). Furthermore, there was no change in schools’ revenue between groups.

**Conclusions:**

Given the evidence of small statistically significant improvements in the energy and saturated fat content, acceptability, and wide reach, this intervention has the potential to influence dietary choices at a population level, and further research is warranted to determine its impact when implemented at scale.

**Trial Registration:**

Australian New Zealand Clinical Trials Registry (ANZCTR) ACTRN12618000855224; https://www.anzctr.org.au/Trial/Registration/TrialReview.aspx?id=375075.

**International Registered Report Identifier (IRRID):**

RR2-10.1136/bmjopen-2019-030538

## Introduction

### Background

Dietary risk factors are a leading cause of disease [1]. Schools are a key setting for public health nutrition [2], and school food outlets can play an important role in children’s nutrition. Studies of Australian school canteens show energy-dense, nutrient-poor foods are the most commonly purchased items [3,4], despite the existence of interventions to encourage healthier choices [5-7]. Such interventions typically include supply-based approaches that target the relative availability of healthier foods. For example, the New South Wales (NSW) Healthy School Canteen Strategy (the NSW Strategy) requires that canteen menus have at least 75% *everyday* items (healthy foods consistent with the Australian Dietary Guidelines) and no *should not be sold* items (unhealthy items high in saturated fat, sugars, or salt) [8]. The NSW Strategy is mandatory in government schools and is strongly encouraged in nongovernment schools [8]. However, the impact of such supply-based interventions is highly variable due to less than optimal implementation [5-7,9,10].

The rise of web-based technology represents a unique opportunity to provide broader support to consumers to encourage healthy canteen purchases in schools using demand-based or consumer-focused approaches. In particular, choice architecture strategies [11], including menu labeling [12-16], prompts [17-19], and changing the position of food [20], have been shown to influence food choices in schools and other settings. Embedding these strategies within routinely used systems is appealing from a public health perspective as they provide the opportunity to reach many people simultaneously at low cost, require only minimal engagement, and are not reliant on the education or skills of the consumers [11].

We previously evaluated a choice architecture intervention delivered via an existing, routinely used web-based canteen ordering system in Australian schools. The pilot study, conducted in 10 NSW government primary schools, established that choice architecture strategies could be successfully embedded within a web-based canteen ordering system [21]. Web-based ordering allows parents and carers (hereafter *parents*) and students to view the school canteen menu and prepurchase items on the web. This cluster randomized controlled pilot trial found that a 2-month intervention, which incorporated menu labeling, positioning, prompting, and availability strategies, significantly increased healthy food purchases [21]. At follow-up, the lunch orders from the intervention schools contained less energy, saturated fat, and sodium than lunch orders from control schools (*P*<.001) [21]. However, the pilot study tested only a limited range of strategies within a small number of government schools for a short period. Furthermore, since the pilot, the NSW canteen guidelines have changed [22]. In 2017, the Fresh Tastes at School Strategy [22], which adopted a traffic light system to classify foods as *green*, *amber*, or *red* from most to least healthy, was replaced by the current NSW Healthy School Canteen Strategy [8], which was revised to align with the Australian Dietary Guidelines. The current NSW Strategy uses the national Health Star Rating front-of-pack labeling system and specific portion limits to classify foods as *everyday*, *occasional*, or *should not be sold* (*caution*). Schools were provided with support to help implement the guidelines, with the goal of complete implementation in NSW schools by 2019.

### Objectives

This study seeks to extend the impact of the pilot by testing additional strategies in a changed policy context with schools from additional sectors (ie, Catholic and independent schools) for a longer period (approximately 12 months) to determine the broader utility of this novel approach in improving public health nutrition. This trial aims to assess the effectiveness of a multistrategy behavioral intervention embedded within an existing web-based canteen ordering system in reducing the energy, saturated fat, sugar, and sodium content of primary school students’ web-based lunch orders.

## Methods

### Study Design

This study used a parallel-group, cohort, cluster randomized controlled trial (RCT) design and is reported according to the CONSORT (Consolidated Standards of Reporting Trials) extension for clustered RCTs. This trial was prospectively registered (ACTRN12618000855224) and approved by the University of Newcastle Human Research Ethics Committee (H-2017-0402) and the Catholic Education Office Dioceses of Sydney, Parramatta, Lismore, Maitland-Newcastle, Bathurst, Canberra-Goulburn, Wagga Wagga, Wollongong, and Wilcannia-Forbes. Schools (clusters) with a web-based canteen ordering system were randomly assigned to either an intervention group (receiving choice architecture strategies embedded in the web-based systems, plus audit and feedback) or a control group (receiving the standard web-based canteen only), as it was not possible to randomize individuals. A detailed protocol has been published [23], which is summarized below.

### Participant Recruitment

#### Participating Schools

Nongovernment (Catholic and independent) primary schools within NSW, Australia, were approached to participate by mail and telephone. Although it was originally intended that government schools would be included, extensive delays in obtaining ethical approval meant that these schools were excluded, as the timeframe of intervention exposure would have been too short to warrant inclusion. Although the NSW Healthy School Canteen Strategy is mandatory in government schools, all schools are strongly encouraged to adopt this strategy [8].

Recruitment took place from May to September 2018 (17 weeks).

#### Inclusion and Exclusion Criteria

Textbox 1 describes the inclusion and exclusion criteria.

**School, user, and order inclusion and exclusion criteria.**

**Inclusion Criteria**

- Schools: schools were eligible if they used the Flexischools web-based canteen service and had done so for at least a month before recruitment. Flexischools is the largest provider of web-based canteen services in Australia, servicing more than 1200 schools and processing more than 13 million lunch orders per year [21].
- Users: students from kindergarten to grade 5 who placed a web-based lunch order in the baseline data collection period were included in the study.
- Orders: all new orders placed on a mobile device were included (typically representing approximately 70% of all orders). Orders placed on a desktop or where order modality could not be determined were excluded as users would not have been exposed to any of the intervention strategies.

**Exclusion Criteria**

- Schools
  Externally licensed school canteens were excluded as these private operators often service multiple schools, increasing the risk of intervention contamination. However, two recruited canteens that were not initially identified as being externally licensed were retained, as they did not service any other school within the sample.
  Combined primary and secondary schools were ineligible unless they had separate menus for primary and secondary students, as the New South Wales Strategy is applied differently across these age groups.
- Users: grade 6 and grade 5 and 6 composite students were excluded from the baseline as they would have moved on to secondary school during the follow-up period. School staff members were excluded.
- Orders: the baseline and follow-up periods were intended to run for the 10-week school term. However, orders from 2 weeks of the baseline term were excluded as the order mode (mobile vs desktop) could not be determined because of a software update. The follow-up data collection period was subsequently reduced to 8 weeks to match the baseline. Recurring orders placed before the intervention period and orders from special food days were excluded, as users would not have been exposed to the intervention strategies. Lunch orders with an implausible number of items (ie, 15 or more) were excluded based on consensus from research dietitians with extensive canteen experience.

### Intervention

#### Overview

**Figure 1:**
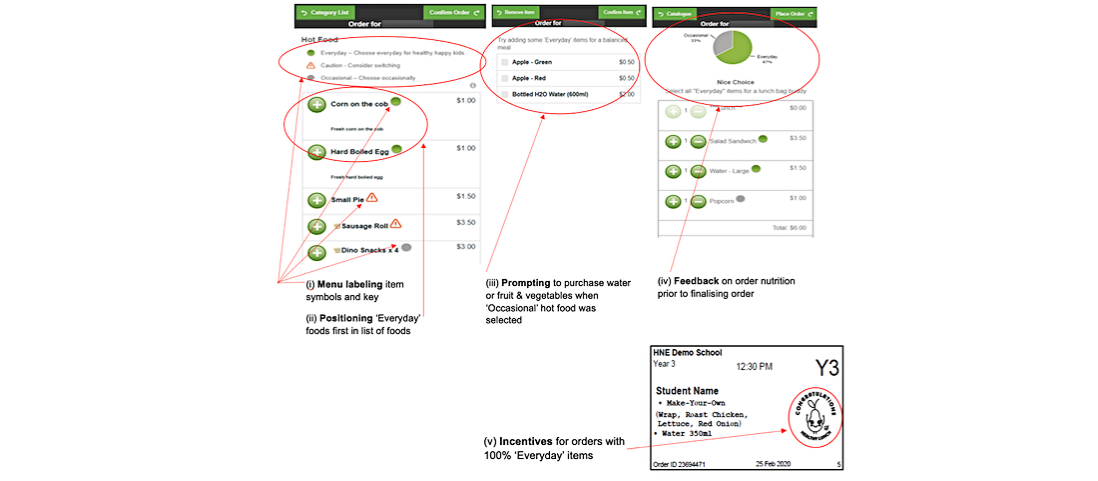
Screenshot of the Click & Crunch intervention.

A multistrategy behavioral intervention targeting users of the web-based system was implemented by modifying the interface of the Flexischools web-based ordering system. The intervention (Figure 1) is fully described in the published protocol [23] and incorporated choice architecture strategies [11], including the following:

- Menu labeling: a colored symbol denoting *everyday*, *occasional*, or *caution* (also referred to as *should not be sold*) items based on the NSW Healthy Canteen Strategy was added next to each menu item along with a key explaining each of the symbols.
- Positioning: menu items and categories were arranged to make healthier *everyday* options more prominent (ie, positioned first within the list of menu items), and where there were multiple flavors of an *occasional* or *caution* item, users were required to *click* on the category before the full list of flavors was displayed in a separate pop-up box.
- Prompting: an appealing image and brief text prompt were placed next to all healthy categories. When users selected an *occasional* or *caution* hot food item, they received a prompt to purchase water and a piece of fruit or vegetable (*healthy add-ons*).
- Feedback: before finalizing and paying for their lunch order, the user was shown a pie graph with tailored feedback based on the proportion of *everyday* items in the order.
- Incentives: orders that contained 100% *everyday* items had a cartoon character and congratulatory text printed on the lunch label, which was printed out and stuck on the paper bag in which the lunch order was delivered to students.

#### Canteen Supportive Strategies

An audit and feedback report were emailed to canteen managers and principals, classifying each menu item as per the NSW Strategy (ie, *everyday* or *occasional*) and providing general information about pricing items to encourage healthy purchases [6].

### Control

Control schools received no change to their web-based canteen menu and no audit or feedback reports. However, NSW Local Health Districts support all schools to use the Menu Check Service—a free service that reviews canteen menus against the NSW Strategy and provides feedback to schools.

#### Outcomes and Measures

##### Overview

Student purchasing data automatically collected by the Flexischools system was used as the basis for evaluation. Data were collected simultaneously for all schools, for two 8-week periods, 12 months apart (term 2, 2018, and term 2, 2019). Baseline data were retrospectively collected.

##### Primary Outcomes

The primary outcomes were the mean lunch order content of energy (kg), saturated fat (g), sugar (g), and sodium (mg). For prepackaged foods, this was based on a blinded dietitian’s assessment using a series of sources in the following order: (1) a database containing more than 2000 commonly stocked canteen products developed by canteen researchers for the past 5 years, (2) the *Healthy Food Finder* database (NSW Government) [24], (3) the *FoodSwitch* website (The George Institute for Global Health) [25], and (4) a web search for the nutrient information panel. For canteen-prepared food, the recipe was obtained from the canteen manager and analyzed using *FoodWorks 9 Professional* nutrition analysis software (Xyris Software) [26].

##### Secondary Outcomes

#### Healthier Purchasing

The proportion of all web-based lunch order items that were *everyday*, *occasional*, and *caution* was calculated by a dietitian using the criteria underpinning the NSW Strategy; the mean proportion of energy within lunch orders that was derived from saturated fat and sugar was also calculated based on 37 kJ/g of fat and 17 kJ/g of sugar [27].

#### Canteen Revenue (Adverse Outcome)

Purchasing data that were automatically collected by the web-based system were used to calculate mean weekly canteen revenue.

##### Canteen Characteristics

At the end of the follow-up period, a telephone interview with canteen managers was conducted to collect information regarding canteen operations (eg, principal operated; parent and citizen operated; or externally licensed—privately operated), type of manager (eg, paid or volunteer manager), the days of operation, and the usual number of web-based orders per week.

##### Process Measures

###### Change in Availability

The proportion of schools with canteen menus meeting the NSW Strategy was calculated at baseline and follow-up based on dietitian menu assessment.

###### Change in Pricing

The average price of *everyday*, *occasional*, and *caution* items was calculated at baseline and follow-up based on each school’s web-based menu.

###### Intervention Acceptability

During the telephone interview, canteen managers were asked to rate the acceptability of the intervention (eg, individual strategies and the intervention overall) using a Likert scale (Multimedia Appendix 1).

###### Additional Support Received

To determine the potential impact of canteen managers making changes to their menu, they were also asked about any additional support they had received during the 12-month intervention period.

##### School Characteristics

School characteristics were obtained from a national website [28]. The following information was extracted: the number of student enrolments, the proportion of Aboriginal and Torres Strait Islander enrolment, and the school postcode.

##### User Characteristics

User characteristics were derived from automatically collected data within the Flexischools system. *Student class* (eg, 3F) contains student grade (eg, Grade 3) and is a required field entered by the parents at system registration, and the frequency of use was based on a count of all orders placed by a student within the baseline data collection period and an average per week was calculated.

#### Randomization and Blinding

Following the provision of principal (cluster) consent, and after the completion of the baseline menu assessment, an independent statistician used Microsoft Excel to randomize schools to an intervention or control group in a 1:1 ratio, using block randomization with block size between 2 and 4. Randomization was stratified by school sector (ie, independent schools and Catholic schools) and socioeconomic status based on school postcode using Socioeconomic Indexes for Areas (SEIFA) [29]. Due to the difficulty in blinding participants, this was run as an open trial.

#### Fidelity and Data Quality

During each school term for the duration of the intervention, a research assistant checked the web-based menus to ensure that the menu labels were correctly applied and recorded whether other intervention strategies were present or absent. An exception was the incentive strategy, which, being printed on lunch labels, was not verifiable by checking the web-based menu. Canteen visits were conducted in 6 schools (including 3 intervention schools) to verify the automatically collected purchasing data against the orders received and distributed by the canteens.

#### Sample Size

Recruitment of 26 schools and 194 students per school (allowing for 86% follow-up and 70% of orders placed using a mobile device) would allow detection of a between-group difference of 195 kJ per average lunch order, assuming an intraclass correlation coefficient of 0.05, with 80% power, and a significance level of *P*<.0125 (Holm-Bonferroni adjusted for the four primary outcomes).

#### Analysis

An intention-to-treat approach was adopted, whereby all student orders and schools were analyzed based on the groups to which they were originally allocated and included data from all students who had baseline purchasing data. To adjust for multiple outcomes, *P*<.0125 was adopted as the prespecified level of significance.

##### Primary Outcomes

Each primary trial outcome was assessed using a separate linear mixed model. The nutritional content (ie, energy, saturated fat, sugar, and sodium) of all web-based lunch orders placed by students was compared between the intervention and control groups throughout time by including a group-by-time interaction fixed effect. All models included a random intercept for school (to account for potential school-level clustering), a nested random intercept and random time effect for students (to account for repeated measurements between and within baseline and follow-up), and fixed effects for the school sector and SEIFA.

##### Secondary Outcomes

Healthier purchasing outcomes (ie, *everyday*, *occasional*, and *caution*) were assessed using separate logistic mixed models. To assess whether there was a significant change in the purchase of *everyday*, *occasional*, and *caution* items between groups, three separate logistic regressions were used (ie, items that are *everyday* vs items that are not *everyday*), including a group-by-time interaction fixed effect. As with the primary outcomes, all models included a random intercept for school (to account for potential school-level clustering), a nested random intercept and random time effect for students (to account for repeated measurements between and within time points), and fixed effects for the school sector and SEIFA. Differences in the proportion of energy derived from saturated fat and sugar within each order and differences in average weekly revenue were assessed according to the primary outcomes.

##### Per-Protocol Analysis

A per-protocol analysis was conducted to determine the effect on energy content (kJ) and proportion of *everyday* foods when the intervention was applied in full. Schools were included if they had >80% of verifiable strategies correctly applied at follow-up and if the incentive strategy was reported as present in the canteen manager survey. Prespecified subgroup analyses were conducted based on energy content (kJ), student grade (kindergarten-grade 2 vs grade 3-5), school sector (Catholic vs independent), and order frequency (*low* <1 order/week on average vs *high* ≥1 order/week on average) by adding a three-way interaction fixed effect (group-by-time-by-subgroup). The following assumptions underpin the prespecified subgroup analyses: (1) parents may have more control over the lunch orders of younger students and may be more influenced by the intervention, leading to healthier purchasing for younger students. (2) There may be differences in the implementation of the NSW Healthy Schools Canteen Strategy between the school sectors (Catholic vs independent), which may have influenced the menu composition, and therefore the ability of the intervention to have an effect. (3) Users who ordered less frequently may consider canteen lunch orders to be more of a *treat* purchase rather than part of their usual diet and therefore be less influenced by the intervention strategies.

Statistical analyses were performed using the SAS version 9.3 (SAS Institute).

## Results

### Overview

**Figure 2:**
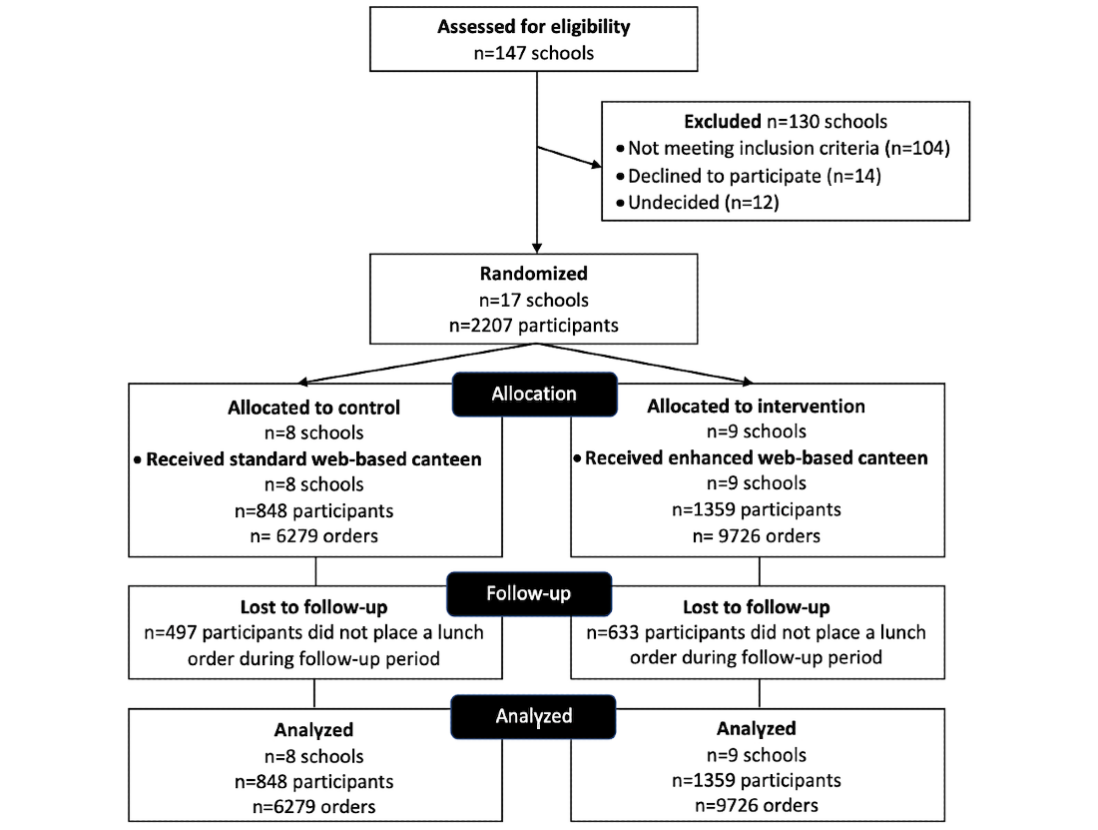
CONSORT (Consolidated Standards of Reporting Trials; extension for clustered randomized controlled trials) diagram.

The CONSORT diagram (Figure 2) shows the number of schools and students participating in the trial. After 17 weeks of recruitment, 40% (17/43) of eligible schools had consented, 33% (14/43) had refused, and 28% (12/43) were undecided. In total, 9 schools were randomized to the intervention group, and 8 schools were randomized to the control group. None of the schools dropped out of the study. Four *combined* schools that enrolled kindergarten to grade 12 students were included in the sample (3 intervention schools and 1 control school).

Only four orders, representing <0.01% of all orders, were excluded because they were implausibly large. The characteristics of participating schools and students are described in Tables 1 and 2. Intervention schools had approximately 30% higher student enrolments than control schools (no significance testing) [30], and as such, the average number of web-based lunch orders per week was higher in intervention schools.

**Table 1:** Characteristics of the sample of 17 participating schools.

School and canteen characteristics		Intervention (n=9 schools)	Control (n=8 schools)
**School sector^a^, n (%)**			
	Independent	4 (44)	3 (38)
	Catholic	5 (56)	5 (63)
Number of enrolments^a,b^, mean (SD)		501 (208)	386 (134)
Aboriginal or Torres Strait Islander students^a^ (%), mean (SD)		6 (9.7)	4 (3.5)
**Socioeconomic status^c^, n (%)**			
	Least advantaged	3 (33)	4 (50)
	Most advantaged	6 (67)	4 (50)
**Type of canteen operation^d^, n (%)**			
	Principal or school run	6 (86)	6 (75)
	Parent & Citizen or Parents & Friends run^e^	0 (0)	1 (13)
	Contracted food service	1 (14)	1 (13)
**Type of canteen manager^d^, n (%)**			
	Paid	7 (100)	8 (100)
	Volunteer	0 (0)	0 (0)
**Days of canteen operation per week^d^, n (%)**			
	5	6 (86)	5 (63)
	3-4	1 (14)	2 (25)
	1-2	0 (0)	1 (13)
Number of weekly web-based lunch orders (per school)^f^, mean (SD)		136 (80.3)	98 (91.3)

^a^On the basis of publicly available school statistics (MySchool 2018).

^b^Excluding combined schools (as this information was not available on the MySchool website).

^c^Socio-economic Indexes for Areas 2016 data, based on the postcode of school locality, and dichotomized (median split).

^d^On the basis of the canteen manager survey conducted after collecting follow-up data, completed by 7 intervention and 8 control canteen managers.

^e^Parent & Citizen or Parents & Friends (in Catholic schools) run canteens are those that are managed by a governing body or committee consisting of parents and citizens of the school community and the school principal.

^f^On the basis of Flexischools purchasing data.

**Table 2:** Characteristics of the sample of 2207 participating students.

User characteristics			Intervention (n=1359)		Control (n=848)
**Grade of student at baseline, n (%)**					
	Kindergarten-grade 2	677 (49.82)		446 (52.59)	
	Grade 3-5	682 (50.18)		402 (47.41)	
**Frequency of use^a^, n (%)**					
	High users (≥1 order/week on average)^b^	463 (34.07)		312 (36.79)	
	Low users (<1 order/week on average)	896 (65.93)		536 (63.21)	

^a^Frequency of use based on baseline purchasing characteristics.

^b^Orders ≥8 during the 8-week baseline data collection period.

### Primary Outcomes

#### Overview

The linear mixed model analysis indicated that the between-group differences throughout time for the intervention group were as follows: −69.4 kJ energy (95% CI −119.6 to −19.1; *P*=.01), −0.6 g saturated fat (95% CI −0.9 to −0.4; *P*<.001), −32.1 mg sodium (95% CI −56.3 to −7.9; *P*=.013), and +0.4 g sugar (95% CI −0.7 to 1.5; *P*=.47). The differences in energy and saturated fat were statistically significant at the prespecified level of *P*<.0125, and sodium was borderline (Table 3).

**Table 3:** Primary and secondary outcomes in intervention and control groups from baseline to follow-up for 2207 student participants (linear mixed model analysis).

Variable		Baseline, mean (SD)		Follow-up, mean (SD)		Intervention versus control^a^			
		Intervention (N=1359 children; N=9726 orders; N=23,526 items)	Control (N=848 children; N=6279 orders; N=14,124 items)	Intervention (N=1108 children; N=9434 orders; N=22,061 items)	Control (N=691 children; N=6334 orders; N=14,087 items)	Main analysis		Per-protocol analysis	
						Group-by-time differential effect (95% CI)	*P* value	Group-by-time differential effect (95% CI)	*P* value
**Primary outcomes^b^**									
	Energy (kJ)^b^	1634.4 (704.2)	1632.1 (743.0)	1623.3 (699.2)	1685.6 (838.6)	−69.4 (−119.6 to −19.1)	.*01*^c^	−89.4 (−148.9 to −29.9)	*.007*
	Saturated fat (g)^b^	5.2 (3.9)	4.6 (3.2)	4.7 (3.7)	4.9 (3.4)	−0.6 (−0.9 to −0.4)	*<.001*	−0.7 (−1.1 to −0.4)	*<.001*
	Sugar (g)^b^	12.9 (14.0)	15.8 (19.1)	13.3 v(14.5)	15.4 (21.1)	0.4 (−0.7 to 1.5)	.47	0.7 (−0.6 to 2.0)	.28
	Sodium (mg)^b^	596.1 (343.0)	599.3 (328.9)	580.1 (342.0)	618.1 (350.7)	−32.1 (−56.3 to −7.9)	.013	−29.9 (−58.1 to −1.8)	.04
**Secondary outcomes^d^**									
	Energy from saturated fat^b^ (%)	11.0 (5.9)	9.9 (5.1)	10.2 (5.8)	10.4 (5.2)	−0.9 (−1.4 to −0.5)	*<.001*	−1.1 (−1.6 to −0.5)	*<.001*
	Energy from sugar^b^ (%)	12.0 (11.8)	13.9 (12.7)	12.4 (11.9)	13.1 (12.7)	1.1 (0.2 to 1.9)	.02	1.5 (0.5 to 2.5)	*.006*
	Average weekly revenue per school (US $)	668.60 (420.90)	496.10 (442.63)	938.60 (574.07)	700.81 (480.06)	65.28 (−76.02 to 206.58)	.36	119.7 (−20.94 to 260.40)	.10

^a^Data were analyzed using separate linear mixed models adjusted for Socioeconomic Indexes for Areas, the school sector, and clustering at the school and student levels.

^b^Baseline intraclass correlation coefficient values: energy 0.100; saturated fat 0.130; sugar 0.131; sodium 0.111.

^c^Italics indicate statistical significance *P*<.0125.

^d^Baseline intraclass correlation coefficient values: percentage energy from sugar 0.104; percentage of energy from saturated fat 0.117.

#### Per-Protocol Analysis

In total, 4 schools partially implemented the intervention, with 3 schools not implementing the healthy add-ons strategy (see the section *Fidelity Checks*), and 4 schools where it could not be confirmed that the incentive strategy was delivered. The per-protocol analysis of the 5 intervention schools that implemented the intervention in full, relative to control schools, showed larger significant effects for three of the four primary outcomes (−89.4 kJ energy, *P*=.007; −0.7 g of saturated fat, *P*<.001; Table 3).

### Secondary Outcomes

#### Healthier Purchasing

From baseline to follow-up, relative to control schools, intervention schools had greater odds of having *everyday* items purchased (odds ratio [OR] 1.69, 95% CI 1.46-1.96; *P*<.001) corresponding to a 9.77% increase in *everyday* items, and lower odds of having *occasional* items purchased (OR 0.67, 95% CI 0.57-0.78; *P*<.001) corresponding to a 7.69% decrease in *occasional* items (Table 4). The decrease in *caution* items was not significant at the prespecified significance level (OR 0.82, 95% CI 0.68-1.00; *P*=.05). The per-protocol analysis revealed that the intervention effect and significance remained similar for both *everyday* (OR 1.50, 95% CI 1.27-1.78; *P*<.001) and *occasional* (OR 0.70, 95% CI 0.59-0.84; *P*=.001) items. There were very small between-group differences in the percentage of energy from saturated fat (−0.9%, 95% CI −1.4% to −0.5%; *P*<.001) and percentage of energy from sugar (+1.1%, 95% CI 0.2%-1.9%; *P*=.02) with the result for energy significant in the main analysis, and both energy and sugar significant in the per-protocol analysis (Table 3).

**Table 4:** Secondary outcomes in intervention and control groups from baseline to follow-up (logistic mixed model analysis; analysis of 73,798 items purchased)^a^.

Classification^b^	Baseline, n (%)		Follow-up, n (%)		Main analysis			Per-protocol analysis	
	Intervention (n=23,526)	Control (n=14,124)	Intervention (n=22,061)	Control (n=14,087)	Relative odds ratio (95% CI)	*P* value	Relative odds ratio (95% CI)		*P* value
*Everyday^c^*	7423 (31.55)	5711 (40.43)	8518 (38.61)	5276 (37.45)	1.69 (1.46-1.96)	*<.001^d^*	1.50 (1.27-1.78)		*<.001*
*Occasional^c^*	11,261 (47.87)	6185 (43.79)	9943 (45.07)	6821 (48.42)	0.67 (0.57-0.78)	*<.001*	0.70 (0.59-0.84)		.*001*
*Caution^c^*	4842 (20.58)	2228 (15.77)	3600 (16.32)	1990 (14.13)	0.82 (0.68-1.00)	.05	0.92 (0.74-1.14)		.39

^a^Separate logistic mixed models were used, which included a random intercept for school (to account for potential school-level clustering), a nested random intercept and random time effect for students (to account for repeated measurements between and within time points), and fixed effects for sector and Socioeconomic Indexes for Areas. Variables were dichotomized (eg, *everyday* vs other items).

^b^Baseline intraclass correlation coefficient values: percentage of *everyday* foods 0.07; percentage of *occasional* foods 0.135; percentage of *caution* foods 0.231.

^c^Chicken nuggets are commonly sold in multiple units. Some schools prepackage them (ie, 1 serve=6 nuggets), whereas other schools allow any quantity to be purchased. To account for this difference, this analysis counted the number of nuggets purchased by a single child at 1 purchasing occasion as a single item.

^d^Italics indicate statistical significance *P*<.0125.

#### Revenue

There was no between-group difference in the average weekly revenue from web-based canteen purchases over time (*P*=.36; Table 3).

#### Subgroup Analysis

There were no differences in intervention effectiveness with respect to energy content across student grade, school sector, or frequency of order (Table 5).

**Table 5:** Intervention impact on mean energy content (kJ) of lunch orders from baseline to follow-up: subgroup analysis for 2207 student participants.

Variable		Baseline, mean (SD)			Follow-up, mean (SD)			Intervention versus control^a^			
		Intervention (n=9726 orders)	Control (n=6279 orders)	Intervention (n=9434 orders)		Control (n=6334 orders)	Group-by-time differential effect (95% CI)		*P* value	Group-by-time-by-subgroup differential effect (95% CI)	*P* value
**Student grade**											
	Kindergarten-grade 2	1557.9 (676.4)	1606.8 (758.0)	1551.5 (688.0)		1635.2 (820.1)	−63.9 (−134.4 to 6.6)		.07	Reference	N/A^b^
	Grade 3-5	1708.7 (722.5)	1658.3 (726.3)	1699.7 (703.0)		1734.7 (853.5)	−71.6 (−144.2 to 1.0)		.05	−7.7 (−108.9 to 93.5)	.87
**Frequency of use**											
	Low (<1 order/week)	1719.4 (706.1)	1753.2 (768.4)	1697.0 (714.9)		1723.5 (805.0)	−26.5 (−95.8 to 42.8)		.43	Reference	N/A
	High (1 or more orders/week)	1592.4 (699.5)	1578.5 (725.1)	1562.3 (679.9)		1659.7 (859.9)	−118.9 (−191.9 to −45.9)		*.003^c^*	−92.4 (−193.1 to 8.2)	.07
**School sector**											
	Independent	1569.9 (672.6)	1579.4 (664.9)	1516.6 (661.9)		1601.3 (638.6)	−96.3 (−181.4 to −11.2)		.03	Reference	N/A
	Catholic	1708.6 (731.9)	1654.8 (773.2)	1738.2 (719.8)		1720.2 (905.8)	−33.4 (−97.5 to 30.8)		.28	63.0 (−43.6 to 169.5)	.22

^a^Analysis adjusted for Socioeconomic Indexes for Areas, school sector, and clustering at the school and student levels.

^b^N/A: not applicable; *P* values not applicable for reference values.

^c^Italics indicate statistical significance (*P*<.01).

### Process Measures

#### Change in Availability

At baseline, no intervention schools and 1 control school had menus consistent with the NSW Strategy, and at follow-up, this had changed to 1 intervention school and zero control schools. The proportion of *everyday*, *occasional*, and *caution* menu items was similar between intervention and control menus at both baseline (489/858, 56.9% and 406/694, 58.5% *everyday* items; 138/858, 16.1% and 107/694, 15.4% *occasional* items; 231/858, 26.9% and 181/694, 26.1% *caution* items, respectively) and follow-up (554/876, 63.2% and 424/703, 60.3% *everyday* items; 150/876, 17.1% and 121/703, 17.2% *occasional* items; 172/876, 19.6% and 158/703, 22.5% *caution* items, respectively).

#### Change in Pricing

There was no between-group difference throughout time in the average price of *everyday* (*P*=.54), *occasional* (*P*=.92), or *caution* (*P*=.66) items.

#### Intervention Acceptability

All intervention canteen managers who completed the interview (7/9, 78%) were satisfied and would recommend the intervention to other canteen managers. Almost all agreed that the intervention strategies were acceptable (6/7, 86%; range 6/7, 86% to 7/7, 100%; Multimedia Appendix 1).

#### Additional Support Received

Of the 15 schools (7 intervention and 8 control schools) that completed the interview, 71% (5/7) of intervention schools and 38% (3/8) of control schools reported using the Menu Check Service during the 12-month intervention period. All intervention schools (7/7, 100%) and 75% (6/8) of the control schools reported receiving other menu support.

#### Fidelity Checks

Menu labeling was the basis for all automated strategies (ie, positioning, provision of tailored feedback, and incentives). As such, verifying that the labels were correctly applied indicated that these other strategies were implemented as intended. The proportions of correct labels across all 9 intervention schools were 93.6% (673/719), 94.3% (666/706), 93.4% (657/703), and 95.3% (696/730) for each of the 4 fidelity checks. There were initial issues with applying the *healthy add-ons* strategy, whereby users were overcharged for add-ons when the chosen menu item was ordered in multiples. This strategy was removed from all intervention menus for items commonly ordered in multiples (ie, chicken nuggets). In addition, this strategy caused changes to the usual layout of the production lists that could be used by managers to make up the orders. As a result, 3 intervention schools requested that the *healthy add-ons* strategy be turned off, and 5 of 9 intervention canteen managers verified that incentives were printed on lunch labels of orders with all *everyday* items.

#### Data Quality

In 6 schools, the purchasing data from the provider (Flexischools) was validated against observed data collected from within the school canteen during school visits. There was a 95.8% (767/800) agreement between the data sources.

## Discussion

### Principal Findings

This trial investigated the effect of *Click & Crunch* on the nutritional quality of students’ web-based lunch orders. Intervention orders had significantly lower energy and saturated fat content relative to controls, but there was no significant difference in sugar or sodium content. Encouragingly, there was no impact on canteen revenue, suggesting no adverse intervention effect in this regard. Intervention schools had significantly greater odds of having *everyday* items purchased and lower odds of having *occasional* items purchased, corresponding to a 9.8% increase in *everyday* items and a 7.7% decrease in *occasional* items, respectively.

There was no significant difference in the odds of having *caution* items purchased between groups throughout time. The fact that all foods are classified into one of three categories means that the significant increase in *everyday* foods must happen at the cost of a decrease in other foods. In this case, the decrease is split into a reduction in the other two categories. The point estimates reflect this decrease, but only the reduction in *occasional* foods was significant, given it is the more common option, whereas the reduction in *caution* foods was not significant, given that it is the rarer option and hence there is less power to detect this effect.

Very few studies have tested the effect of delivering nudge strategies via web-based food ordering systems. In a cluster RCT conducted with 4th-7th grade students from a US school, nudge strategies delivered via a web-based ordering system led to a higher selection of fruit, vegetables, or low-fat milk in lunch orders compared with controls receiving no nudge [31]. However, the study was conducted in a single school with only a 2-week follow-up period. A nonrandomized trial of traffic light nutrition labels applied to 53 products across five food categories within a web-based supermarket found no difference in sales in a 10-week period relative to the comparison store [32]. However, a trial of web-based nudging by providing default orders resulted in a higher purchase of whole grains, fruits, and vegetables among 50 participants from New York food pantries, relative to those receiving brief nutrition education before ordering [33].

The patterns of results for this trial are similar to those of the pilot trial, which found significant reductions in the average energy, saturated fat, and sodium content of intervention lunch orders, and an increase in the proportion of healthy items ordered in the 2-month period immediately after the intervention strategies were switched on [21]. However, the effect sizes in the pilot (ie, −572 kJ between-group difference; 22% increase in *healthy* purchases) were larger than in this trial. Methodological differences between the pilot and this trial may account for the apparent differences in effect sizes. Specifically, the pilot trial was conducted for a much shorter period (2-month vs 12-month follow-up) within government schools, using different classifications (*Fresh Tastes @ School*) [22] and a different labeling system (traffic light symbols). Nonetheless, although modest, the effect on the primary outcomes may be meaningful at the population level and should be considered in light of the potential intervention reach. In NSW, 95% of primary schoolchildren access a canteen, with 55% ordering at least weekly and 4% ordering 3 to 5 times a week [34]. As such, many students could potentially benefit from intervention if disseminated broadly through the school system.

This study planned to include 26 schools. Although ethical approval was eventually obtained for government school participation, the differences in the time of exposure to the intervention between the government and nongovernment schools would have been too great to include in this 12-month trial. As such, the final sample included only 17 schools, reducing the precision of the detected effect estimates. Nonetheless, the trial was still able to detect small differences in primary trial outcomes as significant. In total, 14 schools did not consent to participate and 12 were undecided. As the principal was required to provide consent for this canteen-based intervention, it is recommended that future studies evaluate intervention acceptability with a range of stakeholders, including school principals.

### Limitations

Although the intervention and control schools were broadly similar (no significance testing was conducted) [30]_,_ intervention schools had higher enrolments and more lunch orders at baseline. However, factors that are more closely related to the healthiness of student orders (eg, proportion and price of healthy foods available) were similar between groups. The research team did not have any access to individual demographic characteristics beyond student grade, and future research should seek to collect more information regarding user sociodemographic characteristics to allow an analysis of whether the intervention was more effective in certain subgroups. No data were collected from intervention end users regarding intervention acceptability, and findings regarding canteen manager acceptability were based on only 7 participants. The delay in ethics approval meant that the intervention duration varied from 8 to 10.5 months and that government schools were excluded. Previous research demonstrated no differences between government and nongovernment schools in terms of awareness of or current use of web-based canteens [35]. However, there may be differences among school sectors in terms of compliance with the NSW Strategy, which may affect the relative healthiness of canteen menus (ie, the proportion of *caution* foods vs *everyday* foods). Compliance with the strategy may influence the ability of the intervention to have an effect, in that a school compliant with the guidelines at baseline will have less room to show an improvement (ie, a *ceiling effect*). As such, future research with a larger sample of schools should investigate whether there are differences in intervention effectiveness among school sectors and investigate the relationship with strategy compliance. Finally, our analysis is based on purchasing data and not consumption data. However, objectively recorded purchasing data can provide a reasonably accurate estimate of diet quality [36,37].

The strengths of this trial include a rigorous clustered randomized controlled design. The evaluation of the intervention was based on purchasing data automatically collected by the web-based system for more than 2200 students and verified through in-school observations. The nutritional information extracted from the purchasing data was based on rigorous menu assessment, independently verified by 2 dietitians, and based on established nutrition databases. The 12-month follow-up is also a study strength and addresses the limitations of previous trials involving web-based school lunch ordering systems [21,31,38].

### Conclusions

The *Click & Crunch* intervention evaluated in this cluster RCT used existing web-based canteen ordering systems to implement choice architecture strategies to encourage healthier lunch orders for primary school students. Given the evidence of its effectiveness in decreasing the energy content of student lunch orders and increasing the purchase of *everyday* foods, its acceptability among canteen managers, and its wide reach, this intervention has the potential to influence dietary choices at a population level. The intervention may be a useful addition to the suite of strategies available to policymakers to improve child diet, including supply-side interventions encouraging the increased availability of healthier food options and interventions in nonschool settings, including after-school activities, sporting clubs, and the home. Additional research is required to determine if the results are consistent across all school sectors and sustained for a longer period and to determine intervention cost-effectiveness before investigating intervention impact in primary schools when implemented at scale.

## Appendix

Multimedia Appendix 1 Intervention acceptability to canteen managers.

Multimedia Appendix 2 CONSORT-eHEALTH checklist (V 1.6.2).

## Abbreviations

CONSORT: Consolidated Standards of Reporting Trials
NHMRC: National Health and Medical Research Council
NSW: New South Wales
OR: odds ratio
RCT: randomized controlled trial
SEIFA: Socioeconomic Indexes for Areas
