# Supplementary material for: Effectiveness of a Multistrategy Behavioral Intervention to Increase the Nutritional Quality of Primary School Students’ Web-Based Canteen Lunch Orders (Click & Crunch): Cluster Randomized Controlled Trial
Source: J Med Internet Res. 2021 Sep 7;23(9):e26054. doi: 10.2196/26054 (PMC8456336; doi:10.2196/26054)
Supplement: Multimedia Appendix 1 [file jmir_v23i9e26054_app1.docx]

**Appendix 1:** Intervention acceptability to canteen managers

**Response options:** Strongly agree, Agree, Disagree, Strongly disagree, Prefer not to say.

Items marked with * also have response option: Not applicable

| **Question** | **N (%) Strongly agree/agree** |
| --- | --- |
| I was satisfied with the layout of the revised online menu | 7 (100%) |
| I was satisfied with the usability of the revised online menu | 7 (100%) |
| The telephone calls from the research team to assess the canteen menu were acceptable* | 14 (100%) |
| The telephone calls from the research team to classify NEW menu items were acceptable | 7 (100%) |
| The product classifications (i.e. 'Everyday', 'Occasional', 'Should Not Be Sold') in the menu feedback report were acceptable | 6 (85.7%) |
| The pricing information in the feedback report was acceptable | 6 (85.7%) |
| Labelling all menu items as green, grey or red was acceptable | 6 (85.7%) |
| The healthy eating symbols ('Lunch Bag Buddies') for kids on the labels of healthy lunch orders were acceptable* | 5 (100%) |
| The healthy eating prompts on the online menu (e.g. 'Discover new 'Everyday' tastes and flavours') were acceptable* | 5 (100%) |
| The prompts to purchase water, fruit, and/or vegetables when an 'occasional' hot meal was selected were acceptable | 7 (100%) |
| The placement of healthy items at the top of each category was acceptable* | 7 (100%) |
| Displaying the flavours of less healthy items (e.g. chips) on a second screen that users had to click through was acceptable* | 7 (100%) |
| The pie chart that provided users with feedback about the healthiness of their lunch order was acceptable* | 5 (100%) |
| Overall, the intervention was acceptable | 7 (100%) |
| Overall, the intervention had an effect on what was purchased for online lunch orders | 3 (42.9%) |
| Overall, I was satisfied with the intervention | 7 (100%) |
| Overall, I would recommend the 'Click & Crunch' Online Canteens program to other canteen managers | 7 (100%) |
