# Supplementary material for: Effectiveness of a Multistrategy Behavioral Intervention to Increase the Nutritional Quality of Primary School Students’ Web-Based Canteen Lunch Orders (Click & Crunch): Cluster Randomized Controlled Trial
Source: J Med Internet Res. 2021 Sep 7;23(9):e26054. doi: 10.2196/26054 (PMC8456336; doi:10.2196/26054)
Supplement: Multimedia Appendix 2 [file jmir_v23i9e26054_app2.pdf]

CONSORT-EHEALTH Checklist V1.6.2 Report	Manuscript Number	26054
**(based on CONSORT-EHEALTH V1.6), available at [http://tinyurl.com/consort-ehealth-v1-6].**		
**Date completed**		
12/7/2020 0:52:40		
**by**		
Fiona Stacey		
The effectiveness of a multi-strategy behavioral intervention to increase the nutritional quality of primary school students' online canteen lunch orders: The 'Click & Crunch' cluster randomized controlled trial		
TITLE		
**1a-i) Identify the mode of delivery in the title**		
The intervention is a "multi-strategy behavioral intervention"		
**1a-ii) Non-web-based components or important co-interventions in title**		
The intervention is a "multi-strategy behavioral intervention"		
**1a-iii) Primary condition or target group in the title**		
Included in title "increase the nutritional quality"		
ABSTRACT		
**1b-i) Key features/functionalities/components of the intervention and comparator in the METHODS section of the ABSTRACT**		
Yes; "Schools with an online canteen lunch ordering system were randomly assigned to receive either a multi-strategy behavioral intervention which included choice-architecture strategies (menu-labelling, positioning, prompting, feedback and incentives) embedded in the online system (N=9 schools) or a control which received the standard online ordering system only (N=8 schools)."		
**1b-ii) Level of human involvement in the METHODS section of the ABSTRACT**		
Yes; intervention strategies were "choice-architecture strategies (menu-labelling, positioning, prompting, feedback and incentives) embedded in the online system"		
**1b-iii) Open vs. closed, web-based (self-assessment) vs. face-to-face assessments in the METHODS section of the ABSTRACT**		
Described in manuscript text "primary schools within NSW, Australia were approached to participate by mail and telephone".<br>Evaluation is described in abstract "Student purchasing data that were automatically collected by the online system at baseline (Term 2, 2018) and 12-months later (Term 2, 2019) were used to assess trial outcomes".		
**1b-iv) RESULTS section in abstract must contain use data**		
The intervention being tested was embedded within an existing online system that was already in use. The number of children, orders, and items at each time-point is reported in the results section of the manuscript.		
**1b-v) CONCLUSIONS/DISCUSSION in abstract for negative trials**		
The trial did have a significant effect on 2/4 primary trial outcomes, therefore is not negative.		
INTRODUCTION		
**2a-i) Problem and the type of system/solution**		
Yes - see paragraphs 2 & 3 in the introduction.		
**2a-ii) Scientific background, rationale: What is known about the (type of) system**		
Yes - see paragraphs 3 & 4 in the introduction.		
**Does your paper address CONSORT subitem 2b?**		
Yes. "The aim of this trial is to assess the effectiveness of a multi-strategy behavioral intervention, embedded within an existing online canteen ordering system, in reducing the energy, saturated fat, sugar, and sodium content of primary school students' online lunch orders".		
METHODS		
**3a) CONSORT: Description of trial design (such as parallel, factorial) including allocation ratio**		
Yes. "This study used a parallel group, cohort, cluster RCT design "		
**3b) CONSORT: Important changes to methods after trial commencement (such as eligibility criteria), with reasons**		
Yes. "Although it was originally intended that government schools would be included, extensive delays in obtaining ethical approval meant that these schools were excluded, as the timeframe of intervention exposure would have been too short to warrant inclusion".		
**3b-i) Bug fixes, Downtimes, Content Changes**		
Yes, this is reported in the results. "There were initial issues with applying the 'Healthy add-ons' strategy, whereby users were overcharged for add-ons when the chosen menu item was ordered in multiples. This strategy was removed from all intervention menus for items that were commonly ordered in multiples (ie, chicken nuggets)".		
**4a) CONSORT: Eligibility criteria for participants**		
Yes. "Non-government (Catholic and Independent) primary schools within NSW". "Schools were eligible if they used the 'Flexischools' online canteen service and had done so for at least a month prior to recruitment". "Any student from kindergarten through to grade 5 who placed an online lunch order in the baseline data collection period was included in the study."		
**4a-i) Computer / Internet literacy**		
The online ordering system is currently the exclusive method for ordering in many schools. It is not known if there are parents who do not use this system due to computer literacy.		
**4a-ii) Open vs. closed, web-based vs. face-to-face assessments:**		
Yes. "Non-government (Catholic and Independent) primary schools within NSW, Australia were approached to participate by mail and telephone." "Student purchasing data automatically collected by the Flexischools system was used as the basis for evaluation."		
**4a-iii) Information giving during recruitment**		
Yes. "Non-government (Catholic and Independent) primary schools within NSW, Australia were approached to participate by mail and telephone."<br><br>The recruitment and consent process is reported in full in the previously published protocol (Wyse et al 2019 http://dx.doi.org/10.1136/bmjopen-2019-030538).		
**4b) CONSORT: Settings and locations where the data were collected**		
Yes. "Non-government (Catholic and Independent) primary schools within NSW, Australia were approached to participate by mail and telephone." "Student purchasing data automatically collected by the Flexischools system was used as the basis for evaluation."		
**4b-i) Report if outcomes were (self-)assessed through online questionnaires**		
Outcomes were purchasing data automatically collected by the online system. "Student purchasing data automatically collected by the Flexischools system was used as the basis for evaluation."		
**4b-ii) Report how institutional affiliations are displayed**		
Consent was provided at the school level. Affiliations were not displayed to parents or students.		
**5) CONSORT: Describe the interventions for each group with sufficient details to allow replication, including how and when they were actually administered**		
**5-i) Mention names, credential, affiliations of the developers, sponsors, and owners**		
Yes. "Schools were eligible if they used the 'Flexischools' online canteen service and had done so for at least a month prior to recruitment. Flexischools is the largest provider of online canteen services in Australia, servicing over 1,200 schools, and processing over 13 million lunch orders a year [21]."<br><br>Also reported in conflict of interest section "The provider (Flexischools) was selected through a competitive tender process. Flexischools is a commercial organization that provided the online canteen ordering infrastructure to schools that were included in the study. Flexischools had no role in the study design, data analysis, data interpretation or writing of the manuscript."		
**5-ii) Describe the history/development process**		
Yes. A previous pilot study was undertaken and is reported in the manuscript. "We previously evaluated a choice architecture intervention delivered via an existing, routinely used online canteen ordering system within Australian schools. The pilot study, conducted in ten NSW government primary schools, established that choice architecture strategies could successfully be embedded within an online canteen ordering system [21]."		
**5-iii) Revisions and updating**		
This is not applicable for this intervention.		
**5-iv) Quality assurance methods**		
Yes, reported in the results "Menu labelling was the basis for all automated strategies (ie, positioning, provision of tailored feedback and incentives. As such, verifying the labels were correctly applied indicated that these other strategies were also implemented as intended. The proportion of correct labels across all nine intervention schools was 94%, 94%, 93% and 95% for each of the four fidelity checks."		
**5-v) Ensure replicability by publishing the source code, and/or providing screenshots/screen-capture video, and/or providing flowcharts of the algorithms used**		
This is not possible as the intervention was embedded in an existing system. Screenshots and a description of the intervention components has been provided.		

**5-vi) Digital preservation**		
The intervention is not publicly available.		
**5-vii) Access**		
Yes. "Schools were eligible if they used the ‘Flexischools’ online canteen service and had done so for at least a month prior to recruitment. Flexischools is the largest provider of online canteen services in Australia, servicing over 1,200 schools, and processing over 13 million lunch orders a year [21]."		
**5-viii) Mode of delivery, features/functionalities/components of the intervention and comparator, and the theoretical framework**		
Yes. See 'Intervention' section of the Methods.		
**5-ix) Describe use parameters**		
The intervention was applied to all items in the online ordering system.		
**5-x) Clarify the level of human involvement**		
The intervention is reported in full in the previously published protocol (Wyse et al 2019 http://dx.doi.org/10.1136/bmjopen-2019-030538).		
**5-xi) Report any prompts/reminders used**		
There are no prompts or reminders as part of the study.		
**5-xii) Describe any co-interventions (incl. training/support)**		
Yes. "An audit and feedback report was emailed to canteen managers and principals classifying each menu item as per the NSW Strategy (i.e. ‘Everyday’, ‘Occasional’ etc), and providing general information about pricing items to encourage healthy purchases [6]."		
**6a) CONSORT: Completely defined pre-specified primary and secondary outcome measures, including how and when they were assessed**		
Yes. Data is reported in the methods. "Student purchasing data automatically collected by the Flexischools system was used as the basis for evaluation. Data were collected at the same time for all schools, over two 8-week periods, 12-months apart (Term 2, 2018 and Term 2, 2019)." "The primary outcomes were the mean lunch order content of energy (kilojoules), saturated fat (grams), sugar (grams), and sodium (milligrams)." Secondary outcomes were: "The proportion of all online lunch order items that were ‘Everyday’, ‘Occasional’, and ‘Caution’ was calculated by a dietitian using the criteria underpinning the NSW Strategy; The mean proportion of energy within lunch orders that was derived from: saturated fat; and sugar".		
**6a-i) Online questionnaires: describe if they were validated for online use and apply CHERRIES items to describe how the questionnaires were designed/deployed**		
Not applicable		
**6a-ii) Describe whether and how “use” (including intensity of use/dosage) was defined/measured/monitored**		
The number of children, orders, and items that are included for each group at each time-point is reported. This can be used to determine the number of orders placed through the online system.		
**6a-iii) Describe whether, how, and when qualitative feedback from participants was obtained**		
Not applicable		
**6b) CONSORT: Any changes to trial outcomes after the trial commenced, with reasons**		
Yes. "Non-government (Catholic and Independent) primary schools within NSW, Australia were approached to participate by mail and telephone." "Student purchasing data automatically collected by the Flexischools system was used as the basis for evaluation."		
**7a) CONSORT: How sample size was determined**		
**7a-i) Describe whether and how expected attrition was taken into account when calculating the sample size**		
Yes, this was included "Recruitment of 26 schools and 194 students per school (allowing for 86% follow-up and 70% of orders placed using a mobile devices)"		
**7b) CONSORT: When applicable, explanation of any interim analyses and stopping guidelines**		
Yes. Data is reported in the methods. "Student purchasing data automatically collected by the Flexischools system was used as the basis for evaluation. Data were collected at the same time for all schools, over two 8-week periods, 12-months apart (Term 2, 2018 and Term 2, 2019)." "The primary outcomes were the mean lunch order content of energy (kilojoules), saturated fat (grams), sugar (grams), and sodium (milligrams)." Secondary outcomes were: "The proportion of all online lunch order items that were ‘Everyday’, ‘Occasional’, and ‘Caution’ was calculated by a dietitian using the criteria underpinning the NSW Strategy; The mean proportion of energy within lunch orders that was derived from: saturated fat; and sugar".		
**8a) CONSORT: Method used to generate the random allocation sequence**		
Yes - "Following provision of principal (cluster) consent, and after the completion of the baseline menu assessment, an independent statistician used Microsoft Excel to randomize schools to an intervention or control group in a 1:1 ratio"		
**8b) CONSORT: Type of randomisation; details of any restriction (such as blocking and block size)**		
Yes - "using block randomization with block size between 2 and 4. Randomization was stratified by school sector (ie, Independent schools, and Catholic schools) and the socioeconomic status based on school postcode using Socio-Economic Indexes for Areas (SEIFA) [29]".		
**9) CONSORT: Mechanism used to implement the random allocation sequence (such as sequentially numbered containers), describing any steps taken to conceal the sequence until interventions were assigned**		
Yes "using a randomised number function in Microsoft Excel in a 1:1 ratio" (reported in the previously published protocol: Wyse et al 2019 http://dx.doi.org/10.1136/bmjopen-2019-030538).		
**10) CONSORT: Who generated the random allocation sequence, who enrolled participants, and who assigned participants to interventions**		
Yes "Following provision of principal (cluster) consent, and after the completion of the baseline menu assessment, an independent statistician used Microsoft Excel to randomize schools to an intervention or control group in a 1:1 ratio"		
**11a) CONSORT: Blinding - If done, who was blinded after assignment to interventions (for example, participants, care providers, those assessing outcomes) and how**		
**11a-i) Specify who was blinded, and who wasn’t**		
Schools and parents were not blinded. "Due to the difficulty in blinding participants, this was run as an open trial."		
**11a-ii) Discuss e.g., whether participants knew which intervention was the “intervention of interest” and which one was the “comparator”**		
Intervention schools were aware of the changes applied to their online system. "Control schools received no change to their online canteen menu and no audit and feedback report."		
**11b) CONSORT: If relevant, description of the similarity of interventions**		
Not applicable		
**12a) CONSORT: Statistical methods used to compare groups for primary and secondary outcomes**		
Yes, clustering was accounted for in the analysis "All models included a random intercept for school (to account for potential school level clustering), a nested random intercept and random time effect for students (to account for repeated measurements between and within baseline and follow-up), and fixed effects for sector and SEIFA."		
**12a-i) Imputation techniques to deal with attrition / missing values**		
An intention-to-treat approach was used "We adopted an intention-to-treat approach whereby all student orders and schools were analyzed based on the groups to which they were originally allocated, and included data from all students that had baseline purchasing data. "		
**12b) CONSORT: Methods for additional analyses, such as subgroup analyses and adjusted analyses**		
A per-protocol and pre-specified sub-group analyses were undertaken "A per-protocol analysis was conducted to determine the effect on energy content (kJ) and proportion of ‘Everyday’ foods when the intervention was applied in full. Schools were included if they had >80% of verifiable strategies correctly applied at follow-up, and if the incentive strategy was reported as present in the canteen manager survey. Pre-specified sub-group analyses were conducted based on energy content (kJ); student grade (kindergarten - grade 2 vs grade 3 – grade 5); school sector (Catholic vs Independent); and order frequency (‘low’ < 1 order/week on average vs ‘high’ ≥ 1 order/week on average), by adding a 3-way-interaction fixed effect (group by time by subgroup). Statistical analyses were performed using Statistical Analysis System (SAS) version 9.3 (SAS Institute, Cary, North Carolina, United States)."		
RESULTS		
**13a) CONSORT: For each group, the numbers of participants who were randomly assigned, received intended treatment, and were analysed for the primary outcome**		
Yes. "A total of nine schools were randomized to the intervention group and eight schools were randomized to the control." At baseline, the intervention group had N=1,359 children; N=9,726 orders; N=23,526 items. At baseline, the control group had N=848 children; N=6,279 orders; N=14,124 items.		
**13b) CONSORT: For each group, losses and exclusions after randomisation, together with reasons**		
Yes. "None of the schools dropped out of the study".		
**13b-i) Attrition diagram**		
This information is not applicable.		
**14a) CONSORT: Dates defining the periods of recruitment and follow-up**		
"Recruitment took place from May to September 2018 (17 weeks). "<br><br>"Data were collected at the same time for all schools, over two 8-week periods, 12-months apart (Term 2, 2018 and Term 2, 2019). "		
**14a-i) Indicate if critical “secular events” fell into the study period**		
Not applicable		
**14b) CONSORT: Why the trial ended or was stopped (early)**		
not applicable		
**15) CONSORT: A table showing baseline demographic and clinical characteristics for each group**		
Reported in Table 1. Characteristics of the sample		
**15-i) Report demographics associated with digital divide issues**		

We did not collect any identifying information about individual participants. We have reported the socioeconomic status and proportion of Aboriginal and Torres Strait Islander enrolments for participating schools.		
**16a) CONSORT: For each group, number of participants (denominator) included in each analysis and whether the analysis was by original assigned groups**		
**16-i) Report multiple “denominators” and provide definitions**		
This is reported in Figure 2. CONSORT diagram.		
**16-ii) Primary analysis should be intent-to-treat**		
"We adopted an intention-to-treat approach whereby all student orders and schools were analyzed based on the groups to which they were originally allocated, and included data from all students that had baseline purchasing data."<br><br>"A per-protocol analysis was conducted to determine the effect on energy content (kJ) and proportion of ‘Everyday’ foods when the intervention was applied in full. Schools were included if they had >80% of verifiable strategies correctly applied at follow-up, and if the incentive strategy was reported as present in the canteen manager survey."		
**17a) CONSORT: For each primary and secondary outcome, results for each group, and the estimated effect size and its precision (such as 95% confidence interval)**		
These statistics are reported in Table 2. Primary and secondary outcomes in intervention and control groups from baseline to follow-up		
**17a-i) Presentation of process outcomes such as metrics of use and intensity of use**		
This is not relevant to this intervention.		
**17b) CONSORT: For binary outcomes, presentation of both absolute and relative effect sizes is recommended**		
Yes. "From baseline to follow-up, relative to control schools, intervention schools had greater odds of having ‘Everyday’ items purchased (OR: 1.7; P<.001) corresponding to a 9.8% increase in ‘Everyday’ items, and lower odds of having ‘Occasional’ items purchased (OR: 0.7; P<.001) corresponding to a 7.7% decrease in ‘Occasional’ items."		
**18) CONSORT: Results of any other analyses performed, including subgroup analyses and adjusted analyses, distinguishing pre-specified from exploratory**		
Yes, a per-protocol and pre-specified sub-group analyses are reported.<br>"The per-protocol analysis of the five intervention schools that implemented the intervention in full, relative to control schools, showed larger significant effects for three of the four primary outcomes (-89.4kJ energy P<.007; -0.7 grams of saturated fat P<.001; -29.9 mg sodium P=.0405) (See Table 2). "<br><br>"There were no differences in intervention effectiveness with respect to energy content across: student grade, school sector or frequency of order (see Table 3). "		
**18-i) Subgroup analysis of comparing only users**		
Yes "The per-protocol analysis of the five intervention schools that implemented the intervention in full, relative to control schools, showed larger significant effects for three of the four primary outcomes (-89.4kJ energy P<.007; -0.7 grams of saturated fat P<.001; -29.9 mg sodium P=.0405) (See Table 2). "		
**19) CONSORT: All important harms or unintended effects in each group**		
Yes, revenue was examined as a potential adverse effect. "There was no between-group difference in the average weekly revenue from online canteen purchases over time (P=.364). "		
**19-i) Include privacy breaches, technical problems**		
Not applicable		
**19-ii) Include qualitative feedback from participants or observations from staff/researchers**		
Not applicable		
DISCUSSION		
**20) CONSORT: Trial limitations, addressing sources of potential bias, imprecision, multiplicity of analyses**		
**20-i) Typical limitations in ehealth trials**		
"Despite intervention and control schools being broadly similar (no significance testing conducted) [30] intervention schools had higher enrolments and more lunch orders at baseline. However, factors that are more closely related to the healthiness of student orders (eg, proportion and price of healthy foods available) were similar between groups. The research team did not have any access to individual demographic characteristics beyond student grade, and no data was collected from intervention end-users regarding intervention acceptability. The delay in ethics approval meant that the intervention duration varied from 8 to 10.5 months and that government schools were excluded. However, previous research demonstrated there are no differences between government and non-government schools in terms of awareness of, or current use of, online canteens [35]. It is important to note that the included choice architecture strategies require only minimal conscious engagement and they do not rely on the knowledge, skills or self-regulatory skills of recipients, which are generally higher in more advantaged populations [11] as typically attend non-government schools. Finally, our analysis is based on purchasing data, not consumption data. However, objectively recorded purchasing data can be a reasonably accurate estimate of diet quality [36-37]. "		
**21) CONSORT: Generalisability (external validity, applicability) of the trial findings**		
**21-i) Generalizability to other populations**		
"Given evidence of its effectiveness, acceptability and wide reach, this intervention has potential to influence dietary choices at a population level. As such, further research is warranted to determine its impact in primary schools when implemented at scale, and to investigate its application in other online food ordering systems, such as secondary schools, workplaces, hospitals, and online-ordered home-delivered food. "		
**21-ii) Discuss if there were elements in the RCT that would be different in a routine application setting**		
The Click & Crunch intervention was evaluated via an existing online ordering system, which could be implemented routinely.		
**22) CONSORT: Interpretation consistent with results, balancing benefits and harms, and considering other relevant evidence**		
**22-i) Restate study questions and summarize the answers suggested by the data, starting with primary outcomes and process outcomes (use)**		
"This trial investigated the effect of ‘Click & Crunch’ on the nutritional quality of students’ online lunch orders. Intervention orders had significantly lower energy and saturated fat content relative to controls, but there was no significant difference in sugar or sodium content. Intervention schools had significantly greater odds of having ‘Everyday’ items purchased, and lower odds of having ‘Occasional’ items purchased, corresponding to a 9.8% increase in ‘Everyday’ items, and a 7.7% decrease in ‘Occasional’ items respectively. There was no impact on canteen revenue. "		
**22-ii) Highlight unanswered new questions, suggest future research**		
"Given evidence of its effectiveness, acceptability and wide reach, this intervention has potential to influence dietary choices at a population level. As such, further research is warranted to determine its impact in primary schools when implemented at scale, and to investigate its application in other online food ordering systems, such as secondary schools, workplaces, hospitals, and online-ordered home-delivered food. "		
Other information		
**23) CONSORT: Registration number and name of trial registry**		
The trial was prospectively registered (ACTRN12618000855224) with the Australian and New Zealand Clinical Trials Registry		
**24) CONSORT: Where the full trial protocol can be accessed, if available**		
https://anzctr.org.au/Trial/Registration/TrialReview.aspx?ACTRN=ACTRN12618000855224<br><br>"A detailed protocol has been published [23] and is summarized below."		
**25) CONSORT: Sources of funding and other support (such as supply of drugs), role of funders**		
"The authors declare they have no conflicts of interest to report. This work is supported by the NHMRC, grant number APP1120233. In-kind support is provided by Hunter New England Population Health and the Hunter Medical Research Institute. RW is supported by a Heart Foundation Postdoctoral Fellowship (ID: 102156). LW receives salary support from a NHMRC Career Development Fellowship (ID: APP1128348) and Heart Foundation Future Leader Fellowship (ID: 101175). Neither the NHMRC nor the Heart Foundation had any role in the design of the study, data collection, analysis or interpretation, or dissemination of findings. The provider (Flexischools) was selected through a competitive tender process. Flexischools is a commercial organization that provided the online canteen ordering infrastructure to schools that were included in the study. Flexischools had no role in the study design, data analysis, data interpretation or writing of the manuscript."		
**X26-i) Comment on ethics committee approval**		
The trial was "approved by the University of Newcastle Human Research Ethics Committee (H-2017-0402), and the Catholic Education Office Dioceses of Sydney, Parramatta, Lismore, Maitland-Newcastle, Bathurst, Canberra-Goulburn, Wagga Wagga, Wollongong, and Wilcannia-Forbes".		
**x26-ii) Outline informed consent procedures**		
The recruitment and consent process is reported in full in the previously published protocol (Wyse et al 2019 http://dx.doi.org/10.1136/bmjopen-2019-030538).		
**X26-iii) Safety and security procedures**		
Purchasing data did not include any parent or child identifying details, and as such, there did not appear to be any reasonable expectation of harm.		
**X27-i) State the relation of the study team towards the system being evaluated**		
"The provider (Flexischools) was selected through a competitive tender process. Flexischools is a commercial organization that provided the online canteen ordering infrastructure to schools that were included in the study. Flexischools had no role in the study design, data analysis, data interpretation or writing of the manuscript."		
